# Expression and characterization of mouse prolactin (mPRL) in CHO dhfr- cells

**DOI:** 10.1186/1753-6561-8-S4-P4

**Published:** 2014-10-01

**Authors:** Miriam Fussae Suzuki, Aline Barros da Silva, João Ezequiel de Oliveira, Fernanda dos Santos Arthuso, Paolo Bartolini, Carlos Roberto Jorge Soares

**Affiliations:** 1 Instituto de Pesquisas Energéticas e Nucleares, IPEN-CNEN/SP, Av. Prof. Lineu Prestes, 2242, São Paulo, SP 05508-000,Brazil

## Background

Prolactin (PRL) is a peptide hormone related to innumerous physiological functions such as lactation, reproduction, osmotic and immune regulation [1]. In order to evaluate new drugs many tests are conducted in mouse models. Use of prolactin from different origin in mouse model can result in differences in biological activities [2]. Only 61% sequence homology is observed between mouse PRL (197 amino acid residues, 22.5 kDa) and human PRL (199 amino acid residues, 23 kDa). Authentic mouse PRL, without initial methionine, expressed in periplasmic fluid of *E. coli *has already been characterized and, as known, it does not present the glycosylated isoform (G-PRL) [3]. About 5-30% of PRL from mammalian pituitaries is glycosylated [1]. Physiologically, nonglycosylated and glycosylated PRL presented differences concerning lactogenic activity, binding affinity and mitogenicity. The objective of this work was to produce mPRL in CHO dhfr-cells, with gene amplification based on methotrexate addition, so to obtain the glycosylated and non-glycosylated forms of mPRL directly secreted into serum free media.

## Methods

The mPRL gene was obtained by PCR reaction from a pUC57 plasmid introducing the human signal peptide and the XbaI restriction enzyme sequence. After purification, the gene was introduced into the pEDdc expression vector. After DNA sequencing and analysis in 1% agarose of the fragments obtained by digestion with the restriction enzymes XbaI, EcoRI, BamHI, and XhoI confirming the correct DNA sequence, Lipofectamine was used to introduce the plasmid pEDdc-mPRL into CHO dhfr- cells. For selection, cells were cultivated in alpha MEM medium without nucleosides with 10% dialyzed fetal bovine serum and antibiotics. Dot blot and Western blotting with rabbit anti-mPRL antisera (NIDDK-NIH, USA) were used for analyzing 48 positive clones, methotrexate being used for gene amplification. Cultivation was carried out in serum free media CHO-S-SFM II (Invitrogen, USA) in 10 cm diameter dishes. Purification was based on two chromatographic steps: Ni(II)-based Immobilized Metal Ion Affinity Chromatography (IMAC) and Gel Filtration Chromatography (Sephacryl™ S100-HR) as described previously for human PRL [4,5].

## Results and conclusions

Construction of pEDdc-mPRL plasmid was confirmed by DNA sequencing and restriction analysis. After selection and amplification by increasing concentrations of methotrexate (from 20 nM to 150 nM), mPRL was synthesized by adherent CHO cells, secreted into the media. Mouse PRL in its authentic form was collected every day. The best clone expressing 4 μg mPRL/10^6 ^cells/mL, which is much higher than bacterial expression (0.1 μg/A_600_/mL) [3], was used for production and purification. For the first time mPRL was expressed in CHO cells, both in nonglycosylated and glycosylated forms, as shown in Western Blotting.
